# Arsenic Trioxide Exerts Antimyeloma Effects by Inhibiting Activity in the Cytoplasmic Substrates of Histone Deacetylase 6

**DOI:** 10.1371/journal.pone.0032215

**Published:** 2012-02-22

**Authors:** Xiaoyan Qu, Juan Du, Chunyang Zhang, Weijun Fu, Hao Xi, Jianfeng Zou, Jian Hou

**Affiliations:** Department of Hematology, The Myeloma and Lymphoma Center, Changzheng Hospital, The Second Military Medical University, Shanghai, China; Virginia Commonwealth University, United States of America

## Abstract

Arsenic trioxide (As_2_O_3_) has shown remarkable efficacy for the treatment of multiple myeloma (MM). Histone deacetylases (HDAC) play an important role in the control of gene expression, and their dysregulation has been linked to myeloma. Especially, HDAC6, a unique cytoplasmic member of class II, which mainly functions as α-tubulin deacetylase and Hsp90 deacetylase, has become a target for drug development to treat cancer due to its major contribution in oncogenic cell transformation. However, the mechanisms of action for As_2_O_3_ have not yet been defined. In this study, we investigated the effect of As_2_O_3_ on proliferation and apoptosis in human myeloma cell line and primary myeloma cells, and then we studied that As_2_O_3_ exerts antimyeloma effects by inhibiting activity in the α-tubulin and Hsp90 through western blot analysis and immunoprecipitation. We found that As_2_O_3_ acts directly on MM cells at relatively low concentrations of 0.5∼2.5 µM, which effects survival and apoptosis of MM cells. However, As_2_O_3_ inhibited HDAC activity at the relatively high concentration and dose-dependent manner (great than 4 µM). Subsequently, we found that As_2_O_3_ treatment in a dose- and time-dependent fashion markedly increased the level of acetylated α-tubulin and acetylated Hsp90, and inhibited the chaperone association with IKKα activities and increased degradation of IKKα. Importantly, the loss of IKKα-associated Hsp90 occurred prior to any detectable loss in the levels of IKKα, indicating a novel pathway by which As_2_O_3_ down-regulates HDAC6 to destabilize IKKα protein via Hsp90 chaperone function. Furthermore, we observed the effect of As_2_O_3_ on TNF-α-induced NF-κB signaling pathway was to significantly reduced phosphorylation of Ser-536 on NF-κB p65. Therefore, our studies provide an important insight into the molecular mechanism of anti-myeloma activity of As_2_O_3_ in HDAC6-Hsp90-IKKα-NFκB signaling axis and the rationale for As_2_O_3_ can be extended readily using all the HDAC associated diseases.

## Introduction

During the last decade, arsenic trioxide (As_2_O_3_) has been demonstrated the efficacy and safety treatment for acute promyelocytic leukemia (APL) [1], [2]. Currently, many trials have represented that the addition of As_2_O_3_ to standard treatment regimens improves survival outcomes in patients and may allow a reduction in cytotoxic chemotherapy exposure in other malignancies, particularly multiple myeloma (MM) and myelodysplastic syndromes (MDS) [3], [4], [5], [6]. Several trials have evaluated the efficacy of As_2_O_3_ in combination with existing MM therapies, including melphalan, dexamethasone, ascorbic acid, and bortezomib, in relapsed patients [3], [4].

Arsenic acts on cells through a variety of mechanisms, influencing numerous signal transduction pathways and resulting in a vast range of cellular effects that include apoptosis induction, growth inhibition, promotion or inhibition of differentiation, and angiogenesis inhibition [7], [8], [9], [10]. In MM, As_2_O_3_ induces apoptosis of MM cells via caspase-9 and overcomes the protective effect of IL-6 in the BM milieu by inhibiting JAK-STAT survival signaling in tumor cells. Moreover, As_2_O_3_ reduces tumor necrosis factor (TNF) α-induced adhesion to bone marrow stromal cells (BMSCs) and the resultant induced secretion of cytokines (IL-6 and VEGF) that promote MM cell growth, survival, and migration [8].

Modification of proteins by histone acetyltransferases (HAT) or histone deacetylases (HDAC) plays an important role in the control of gene expression, and its dysregulation has been linked to myeloma and others malignant transformation or diseases [11], [12]. To date, eighteen HDAC family members (HDAC1-11 and SIRT1-7) have been identified and divided into 5 groups: class I (HDAC1, HDAC2, HDAC3, and HDAC8), class IIa (HDAC4, HDAC5, HDAC7, and HDAC9), class IIb (HDAC6 and HDAC10), class III (SIRT family), and class IV (HDAC11) according to their homology to yeast histone deacetylases. They play an important role in regulating gene transcription as well as a variety of cellular functions [13], [14]. HDAC6, a unique cytoplasmic member of class II, has become a target for drug development to treat cancer due to its major contribution in oncogenic cell transformation [12]. Most of the studies focus on its major substrate α-tubulin and how (de)acetylation of tubulin affects lymphocyte chemotaxis, cellular adhesions, aggresome formation, EGFR signaling, stress granules in stress response, and growth factor-induced actin remodeling and endocytosis [15], [16]. Hsp90 was the second HDAC6 substrate identified in the cytoplasm after α-tubulin [17]. Hsp90 is a molecular chaperone that is induced in response to cellular stress and stabilizes client proteins involved in cell cycle control and proliferative/anti-apoptotic signaling. Chaperone Hsp90 has been described as components of the IKK complex, which associated with its co-chaperone cdc37 behaves as a stabilizing factor of IKK through interaction between cdc37 and the kinase domains of IKKα and IKKβ in NF-κB signaling [18].

Although As_2_O_3_ has been extensively studied as potential anti-myeloma treatment, the precise functions of As_2_O_3_ in the myeloma cells remain to be defined; especially whether As_2_O_3_ could affect activity in HDAC6-Hsp90-IKKα-NFκB signaling axis. Therefore, in this study we examined As_2_O_3_ exerts antimyeloma effects involving in activity toward cytoplasmic substrates α-tubulin and Hsp90 of HDAC6, IKK complex, and then the direct phosphorylation of p65 on NF-κB signaling pathway, which may provide a novel molecular basis and rationale for the use of As_2_O_3_ in MM treatment.

## Results

### As_2_O_3_ inhibits myeloma cell growth and induces apoptosis

**Figure 1:**
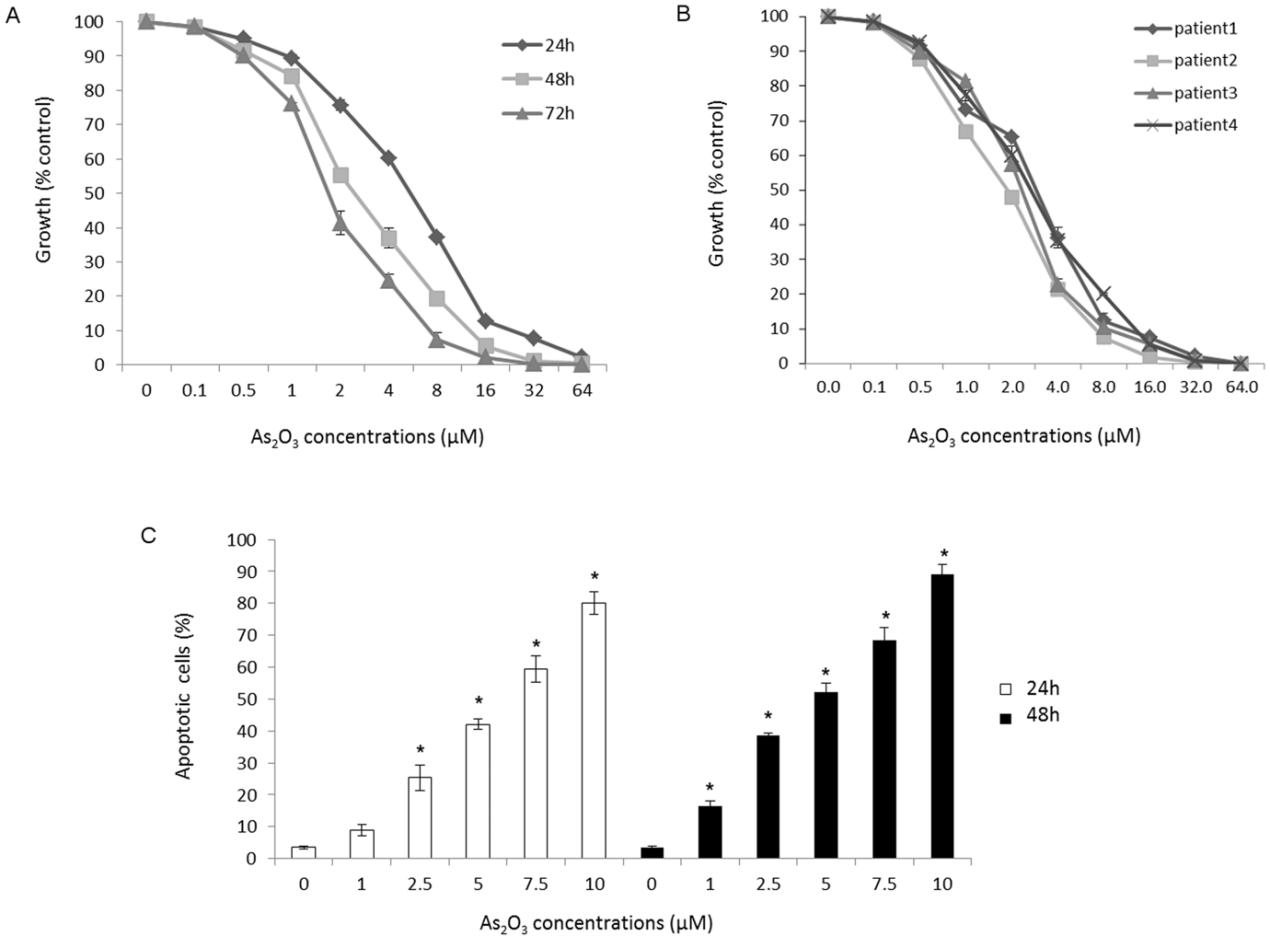
As_2_O_3_ inhibits myeloma cells growth. As_2_O_3_ with different concentrations (0.1 µM∼64 µM) inhibits proliferation of (A) MM cell lines for 24 hours, 48 hours, and 72 hours and (B) MM patients' cells for 48 hours. Cell growth inhibition was measured with the CCK-8 reagent, as described in the Methods. (C) As_2_O_3_ induced apoptosis of NCI-H929 cells. Apoptosis rates were determined using Annexin-V/PI staining. Cells were incubated for 24 or 48 hours with 1 µM∼0 µM of As_2_O_3_, and the appropriate combination and analyzed by flow cytometry. Data are the mean+SD for three replicate measurements. * means statistical difference was observed between the treated group and control (P<0.05).

In order to determine the cell proliferation to As_2_O_3_, MM cell line was treated with drug at the concentration from 0.1 µM to 64 µM for 24, 48 and 72 hours and four primary myeloma cells were cultured with As_2_O_3_ (0.1∼64 µM) for 48 hours (Figure 1). As_2_O_3_ inhibited the growth of MM cells in a dose- and time-dependent manner (Figure 1A). Fifty percent growth inhibition (IC50) in NCI-H929 cells at 48 hours was observed with 2.5 µM As_2_O_3_, and As_2_O_3_ effects in inhibiting cell growth displayed IC50≤4 µM on primary MM cells (Figure 1B). Taken together, the results demonstrate As_2_O_3_ inhibits the proliferation of MM cells at the relatively low concentration both in cell line and primary myeloma cells.

Then we determined the percentage apoptosis of NCI-H929 exposed to As_2_O_3_ by flow cytometry using Annexin V FITC/PI assay as depicted in Figure 1C. There was a gradual increase in Annexin V positive cells (apoptotic cells) in As_2_O_3_-treated cells compared to the control. Treatment of NCI-H929 cells with As_2_O_3_ for 48 hours resulted in 3.1% to 89% apoptosis, respectively, at the dose dependent apoptosis of As_2_O_3_ from 0 µM to 10 µM, with effective dose for 50% apoptosis (ED50) between 2 µM∼5 µM. These results suggest that As_2_O_3_ is a potent inducer of apoptosis in myeloma, particularly at the relatively low concentration.

### As_2_O_3_ decreases HDAC activity in myeloma cells

**Figure 2:**
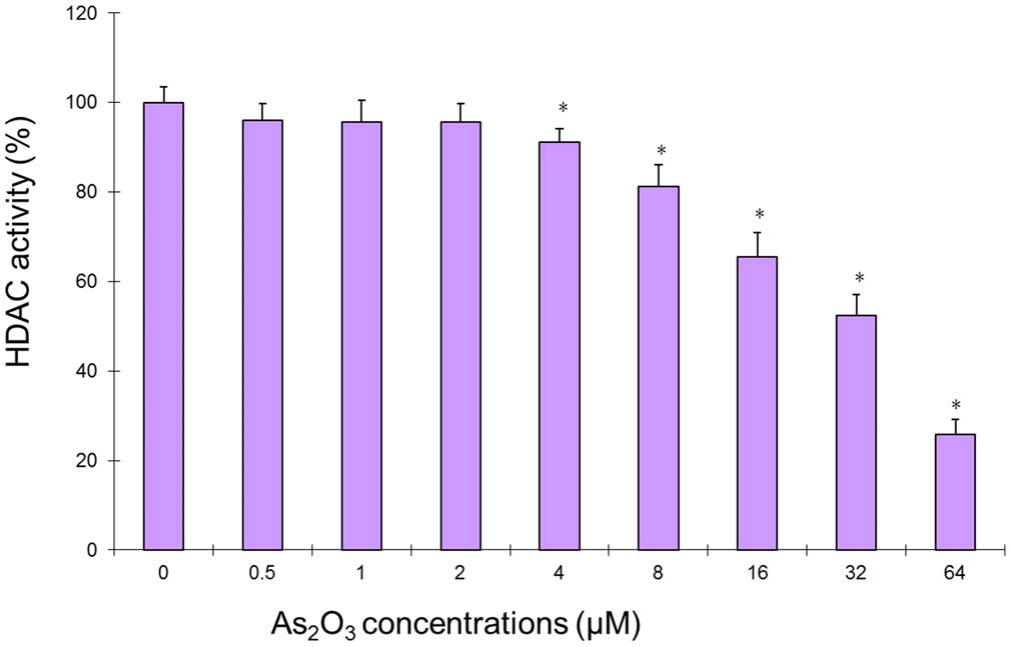
As_2_O_3_ can cause a decrease in HDAC activity. Graphs in the panel are HDAC activity expressed as ODs, in the same scale as the positive and negative controls that are also HeLa cells extracts with and without trichostatin A treatment. At the bottom are values of As_2_O_3_ treatment concentration. * means statistical difference was observed between the treated group and control (P<0.05).

To investigate whether a decrease in histone acetylase activity could be achieved by As_2_O_3_ treatment in the myeloma cells, enzyme activity was evaluated by colorimetric commercial HDAC activity assay in NCI-H929 cells. Results are also shown in Figure 2. Using the same scale for HeLa cell extracts treated or without trichostatin A as positive and negative control, we found that the deacetylase activity was significantly decreased in cells treated with relatively high concentration range from 4 µM∼64 µM (*p*<0.05), however, the deacetylase activity had no change in cells treated with 0.5 µM∼2 µM lower concentrations. When cells treated at the maximum concentration 64 µM in this study, the deacetylase activity was inhibited over 70%, which showed in a concentration-dependent manner.

### As_2_O_3_ triggers the accumulation of α-tubulin acetylation

**Figure 3:**
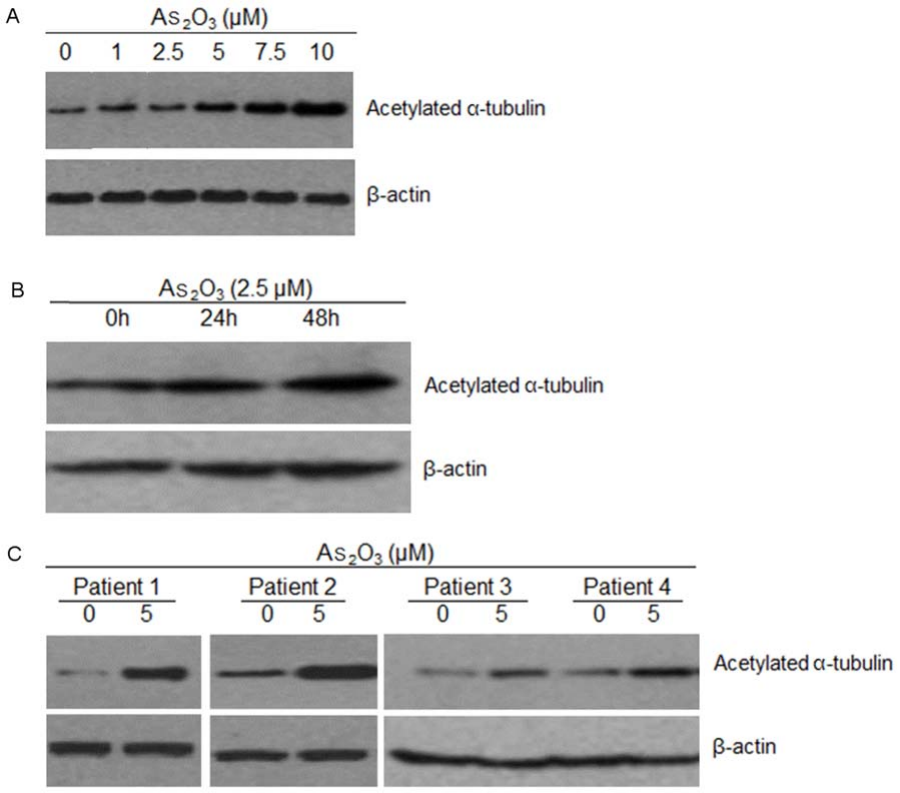
As_2_O_3_ triggers the accumulation of α-tubulin acetylation. (A) NCI-H929 cells were cultured with As_2_O_3_ (1 µM, 2.5 µM, 5 µM, 7.5 µM and 10 µM) for 48 hours. (B) NCI-H929 cells were cultured with As_2_O_3_ (2.5 µM) for the indicated time periods. (C) Primary tumor cells from MM patients were cultured with As_2_O_3_ (5 µM) for 12 hours.

HDAC6 has emerged as a major cytoplasmic deacetylase functioning as α-tubulin deacetylase and Hsp90 deacetylase. To assess the biologic significance of As_2_O_3_ on HDAC6 activity, we next examined the acetylated α-tubulin level in cells. Since 2.5 µM and 5 µM As_2_O_3_ decreased the cell viability to about 50% in cell line and primary cells, we used these concentrations to further study the activity of As_2_O_3_ in myeloma cells. NCI-H929 cells were incubated with As_2_O_3_ from 1 µM to 10 µM for 48 hours, and exposed to As_2_O_3_ at 2.5 µM at 24 and 48 hours, respectively. The whole-cell extracts were then analyzed by western blot. As_2_O_3_ treatment in a dose- and time-dependent fashion markedly increased of acetylated α-tubulin in myeloma cells (Figure 3A and 3B). Importantly, As_2_O_3_ also triggered the accumulation of α-tubulin acetylation in primary tumor cells with 5 µM for 12 hours (Figure 3C).

### As_2_O_3_ induces hyperacetylation of Hsp90 and decreases IKKα-binding to Hsp90

**Figure 4:**
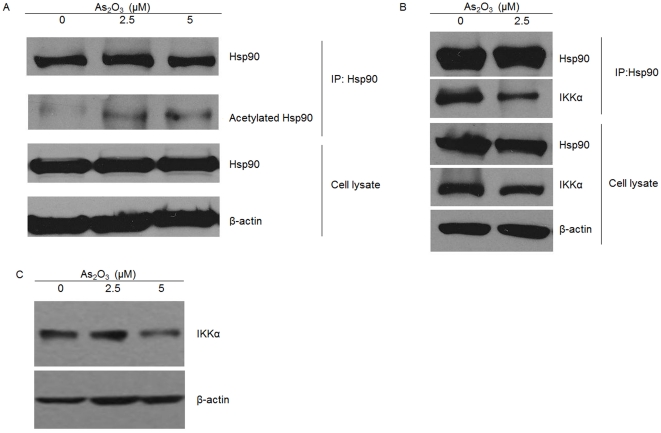
As_2_O_3_ induces hyperacetylation of Hsp90 and decreases IKKα-binding to Hsp90 by immunoprecipitation and western blot assays. (A) The accumulation of acetylated Hsp90 in NCI-H929 cells were cultured with As_2_O_3_ (0 µM, 2.5 µM, and 5 µM) for 48 hours. (B) Hsp90-IKK interaction in NCI-H929 cells were cultured with As_2_O_3_ (2.5 µM) for 48 hours. (C) The IKKα protein expression in myeloma cells treated with As_2_O_3_ (0 µM, 2.5 µM, and 5 µM) for 48 hours.

Next, we determined the second HDAC6 substrate which is the well-characterized chaperone Hsp90. A potential effect of As_2_O_3_ on the Hsp90-IKK interaction was investigated by immunoprecipitation (Figure 4). We observed the greater increased accumulation of acetylated Hsp90 in NCI-H929 cells treated with As_2_O_3_ at 2.5 µM or 5 µM compared to the control (Figure 4A). Since Hsp90 clients are degraded by the ubiquitin-proteasome pathway when Hsp90 function is inhibited [19], we investigated the effect in As_2_O_3_-mediated degradation of IKK in myeloma cells. Binding of Hsp90 to the IKKα was disrupted when cells were treated with As_2_O_3_ 2.5 µM for 48 h, while the total Hsp90 in the input material was not affected and the IKKα protein levels was very mild decreased during the same time point (Figure 4B). Furthermore, we performed western blot assay and observed that the IKKα protein was down expression in myeloma cells treated with As_2_O_3_ 5 µM, whereas there was ineffective with As_2_O_3_ at 2.5 µM (Figure 4C).

### As_2_O_3_ inhibits TNF-α-dependent p65 phosphorylation

**Figure 5:**
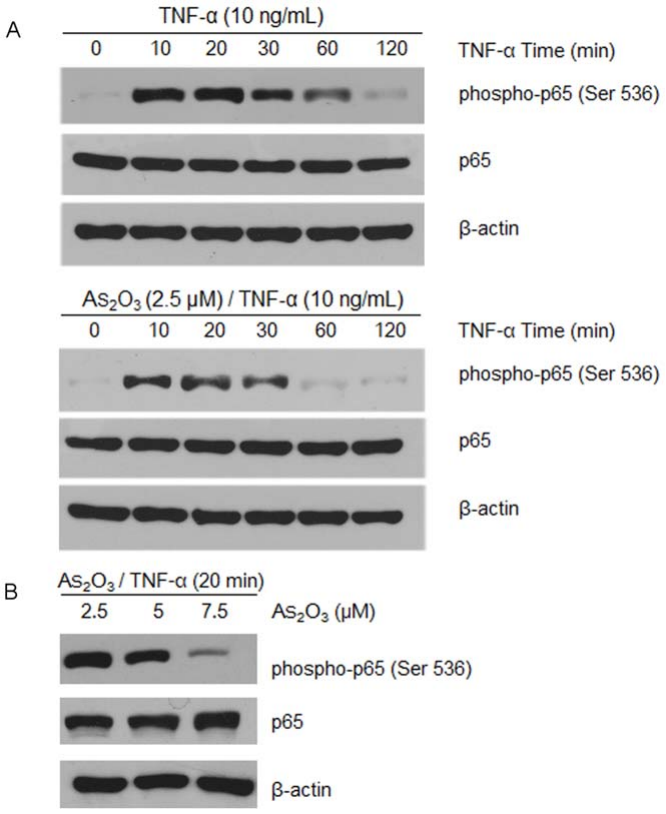
As_2_O_3_ inhibits TNF-α dependent p65 phosphorylation. (A) Cells were either untreated or pretreated with 2.5 µM As_2_O_3_ for 6 hours and then treated with 10 ng/mL TNF-α for 0, 10, 20, 30, 60, 120 minutes time points. (B) Cells were incubated with 2.5 µM, 5 µM, 7.5 µM As_2_O_3_ for 6 hours and then treated with 10 ng/mL TNF-α for 20 minutes. Western blot analysis was performed with anti-p65 and anti-phospho- p65-Ser 536.

To examine the effect of As_2_O_3_ on the NF-κB activation pathway, TNF-α was used, which it is relatively well understood of a potentiated pathway activated agent. The activation of NF-κB involves the phosphorylation, ubiquitination, and degradation of the inhibitor of NF-κB (IκBα), which leads to the nuclear translocation of the p50-p65 subunits of NF-κB followed by p65 phosphorylation, acetylation and methylation, DNA binding, and gene transcription [20]. Phosphorylation of the p65 subunit of NF-κB is required for the transcriptional activation of NF-κB. Stimulus-induced phosphorylation of multiple amino acid residues in the p65 subunit is required for transcriptional activation of NF-κB in various cell types [21], [22]. Since TNF-α has been shown to vary significantly phosphorylation of p65 at Ser 536 in the transactivation domain (TAD) compared to Thr 254, Ser 276, Ser 311, and Ser 529 in a variety of cell types [23], we chose to detect phosphorylation of p65 at Ser 536 in myeloma cells. NCI-H929 cells were pretreated without or with As_2_O_3_ for 6 hours at 2.5 µM, exposed them to 10 ng/mL TNF-α for different times, and examined them for p65 by western blot. We observed that TNF-α induced p65 phosphorylation within 30 minutes, and significantly suppressed the phosphorylation of p65 with the time-dependent in overall interval times, importantly As_2_O_3_ could further suppressed p65 phosphorylation in response to TNF-α treatment in myeloma cells (Figure 5A). Subsequently, we examined the p65 phosphorylation for cells incubated with 2.5 µM, 5 µM, 7.5 µM As_2_O_3_ for 6 hours and then treated with TNF-α for 20 minutes. We found that As_2_O_3_ inhibited the p65 phosphorylation induced by TNF-α in dose-dependent matter (Figure 5B).

## Discussion

In the present study, we show that the molecular mechanisms of the action of As_2_O_3_ against MM by inhibiting activity in the cytoplasmic substrates of HDAC6. We first demonstrate that As_2_O_3_ acts directly on MM cells at low concentrations of 0.5 µM∼2.5 µM, inhibiting the proliferation of myeloma cells at time- and dose-dependent fashion, and As_2_O_3_ induced apoptosis in MM cells, as evidenced by an increase in the annexin V-positive and PI negative apoptotic cell population. These results have been confirmed and extend previous investigations [8], [24], [25]. Subsequently, we observed As_2_O_3_ represses the HDAC activity at the high concentration with 64 µM markedly reduction HDAC activity over 70%. Previous studies also suggested that As_2_O_3_ at much high concentration (great than 500 µM) affects multiple cellular functions via diversely enzyme activity [26], which represses the NF-kB pathway by inhibiting IKK activity at high As_2_O_3_ concentration [27] whereas lower concentrations were ineffective for IKK activity [28]. In several leukemia and lymphoma and solid tumors cells, some studies have reported that the high concentrations of As_2_O_3_ (greater than 10 µM) treatment activated the Jun N-terminal kinase (JNK) and p38, members of stress-activated signal transduction pathways, and resulted in apoptosis [29], [30]. Recently study showed that HDAC inhibitor, scriptaid, induces glioma cell apoptosis through JNK activation and inhibits telomerase activity [31]. Therefore, we speculated that the HDAC activity was significantly decreased by As_2_O_3_ treatment at higher concentrations, which might mediate a stress response involving in JNK activation. Unlike As_2_O_3_ activation at low As_2_O_3_ concentrations (0.5 µM∼2 µM) is correlated with proliferation and apoptosis induction in myeloma cells through radical oxygen species–mediated pathways [32], [33]. Further detailed mechanism analysis of the HDAC activation at higher concentration will be required to verify this hypothesis. In addition, the binding of As_2_O_3_ to critical thiol group in the enzyme, or alternatively, arsenite may alter the structure of the histone deacetylase complex, which might be attributed to HDAC inhibition after high-dose As_2_O_3_ stress treatments.

Class IIb HDAC6 has emerged as a major cytoplasmic deacetylase, which mainly functions as α-tubulin deacetylase and Hsp90 deacetylase [17], thereby regulating cell motility, adhesion and chaperone function. HDAC6 mediates the formation of aggresomes and thus represents a protective cellular response to cytotoxic effects of misfolded protein [34]. Our results showed that treatment of myeloma cells with As_2_O_3_, followed by immunoblotting of the cell extracts with anti-(acetylated tubulin) antibody resulted in markedly increased α-tubulin acetylation in a dose- and time- dependent fashion. Importantly, As_2_O_3_ also triggered the accumulation of α-tubulin acetylation in primary tumor cells. There is evidence that the overexpression of HDAC6 leads to a global deacetylation of α-tubulin, whereas a decrease in HDAC6 increases α-tubulin acetylation [35]. Therefore, we suggested that As_2_O_3_ inhibited HDAC6 through enhanced accumulation of acetylated α-tubulin. Previous studies showed that HDAC6 acetylated tubulin via its tubulin acetylase domain and then resulted in stabilizing the microtubule assembly [35]. We assume that HDAC6-dependent α-tubulin acetylation contributes to the decreased cell motility and invasive migration of myeloma cells; thus, this action of the As_2_O_3_-induced α-tubulin acetylation could be one of the reasons for As_2_O_3_ in myeloma treatment. Several clinical trials have shown the superiority of As_2_O_3_ in myeloma therapy for refractory and relapse MM patients [4], [36].

The second HDAC6 substrate is the well-characterized chaperone Hsp90 [18]. There is evidence that inhibition of HDAC6 results in increased acetylation of Hsp90 and disruption of the chaperone association with its client proteins [37]. IKK is a client of Hsp90 protein complex composed of three subunits, IKKα (IKK1), IKKβ (IKK2) and IKKγ (NEMO) [38]. Previous studies have shown that co-chaperone Cdc37 recruits Hsp90 to the IKK complex in a transitory manner, preferentially via IKKα [18]. We firstly hypothesized that As_2_O_3_ may trigger Hsp90 down-regulation. Our results observed that As_2_O_3_ resulted in accumulation of acetylated Hsp90 in myeloma cells and inhibition of chaperone association with IKKα and increased degradation of IKKα. Importantly, the loss of IKKα-associated Hsp90 occurred prior to any detectable loss in the levels of IKKα, indicating a novel pathway by which As_2_O_3_ down-regulates HDAC6 to destabilize IKKα protein via Hsp90 chaperone function. It has been shown that Hsp90 could be recruited to membrane ruffles, where deacetylated Hsp90 promotes cell motility [39]. Some reports showed that specific inhibition of Hsp90 chaperone function by geldanamycin (GA), an anti-tumor drug, leads to degradation of its clients [19]. Taken together, our results of HDAC6-dependent As_2_O_3_ action may also be mediated through arrest cell motility with Hsp90, which has already emerged as a promising class of anti-cancer drugs in myeloma.

IKK complex directly phosphorylates IkBs (inhibitors of NF-kB) for subsequent proteasomal degradation, which leads to activation of NF-kB, a transcription factor family involved in diverse biological processes [40]. The activation of NF-κB involves the phosphorylation, ubiquitination, and degradation of IκBα and phosphorylation of p65, which in turn lead to the translocation of NF-κB to the nucleus where it binds to specific response elements in the DNA [41]. Phosphorylation of the p65 subunit of NF-κB is required for the transcriptional activation of NF-κB in a number of ways: by stabilizing p65 protein, regulating DNA-binding activity, decreasing the binding of p65 to IκBα and enhancing its transactivation potential [22] .To assess the biologic significance of NF-κB activation during As_2_O_3_-induced growth inhibition, we were designed to investigate the effects of As_2_O_3_ on TNF-α-induced phosphorylation of Ser-536 p65 NF-κB signaling pathway. Ser-536 is located in the COOH terminal TAD of p65 and its phosphorylation plays a key role in transcriptional activation in response to stimuli such as TNF-α [42]. We showed that, in cells treated with As_2_O_3_, phosphorylation of Ser-536 on NF-κB p65 was significantly reduced in response to TNF-α in dose- and time- dependent matter. Consistent with our findings in other types cells [23], TNF-a-induced phosphorylation of Ser-536 on p65 was also significantly reduced in cells, thus attenuating activation of NF-κB signaling.

In conclusion, this study showed for the first time that As_2_O_3_ exerts antimyeloma effects by inhibiting HDAC activity, promoting α-tubulin acetylation, decreasing Hsp90 function, resulting in NF-κB inactivation. Together, the results in the present study elaborated a novel molecular mechanism link among As_2_O_3_ in HDAC actively by inhibiting activity in the cytoplasmic substrates of HDAC6. Thus, our data here thus may provide an important insight into the molecular mechanism of anti-myeloma activity of As_2_O_3_, and the rationale for As_2_O_3_ can be extended readily using all the HDAC associated diseases.

## Materials and Methods

### Cells and reagents

Myeloma cell line NCI-H929 secreting the IgA κ light chain was a gift from Dr. Margaret H.L. Ng (Prince of Wales Hospital, Chinese University of Hong Kong). Cells were cultured in RPMI 1640 medium (Sigma–Aldrich, St. Louis, MO, USA) supplemented with 10% heat-inactivated fetal bovine serum (FBS), 100 U/mL penicillin, 100 µg/mL streptomycin, and 2 mmol/L l-glutamine at 37°C in humidified air containing 5% carbon dioxide. Culture medium was replaced every 3 days. As_2_O_3_ power was purchased from Sigma Co., USA (Lot:A1010) and stored at room temperature, and then it was diluted in culture media just before use. All experiments were conducted with cells in logarithmic phase. TNF-α was purchased from Promega (Madison, WI).

### Antibodies

For Western blot, the following antibodies were used: mouse monoclonal antibodies against Hsp90 antibody (Stressgen Biotechnology), p65 (Santa Cruz, CA), α-tubulin and anti-β-actin (Sigma,USA), and anti–IKKα (Imgenex, San Diego, CA). Phosphospecific anti-p65 (Ser536) antibodies were purchased from Cell Signaling Technology (Danvers, MA). Anti-acetyl-lysine antibodies were from Upstate Biotechnology, Inc. (Lake Placid, NY).

### Primary multiple myeloma cells

Primary myeloma cells were isolated from the bone marrow samples of four MM patients receiving routine diagnostic aspiration, with informed consent approved by the Institutional Ethics Committee. Briefly, cells were separated by Ficoll density gradient centrifugation and washed in phosphate-buffered saline (PBS) twice prior to incubation with an anti-CD138 antibody coupled to magnetic beads (Miltenyi Biotechm Aurburn, CA), and selection using a magnetic affinity column, according to manufacturer's recommendation. Purity of the cell preparation was verified with fluorescence-activated cell-sorting (FACS) analysis and light microscopy to be at >95%. The fresh purified MM cells were cultured for overnight to preactivate in RPMI 1640 medium (GIBCO-BRL, Grand Island, NY), supplemented with 10% FBS, 100 U/mL penicillin, 100 µg/mL streptomycin, and 2 mmol/L l-glutamine (GIBCO-BRL) and IL-6 (20 ng/mL; R&D Systems, Abington, UK) during ex vivo culture.

### Cell viability

Cell viability was tested by colorimetric assay kit (CCK-8 assay kit; Dojindo Laboratories, Tokyo, Japan) based on the MTT assay, according to the manufacturer's instructions. Briefly, 5×10^3^ cells were incubated in 96-well plates with 0.1, 0.5, 1, 2, 4, 8, 16, 32, or 64 µM As_2_O_3_ treatments in culture medium for different time points, and then 10 µL of the CCK-8 solution was added to each well. After 4 hours incubation at room temperature, the optical density (OD) was measured using a spectrophotometer (Molecular Devices Co., Sunnyvale, CA) and the fold-increase in the OD compared to that of the control (proliferation index) was calculated. All experiments were performed in triplicate.

### Detection of Apoptosis

Cell apoptosis was detected by using annexin V staining. MM cells were cultured in media alone, or with media plus with various concentration As_2_O_3_ treatments in culture medium for 24 and 48 hours. Cells were then washed twice with ice-cold PBS and resuspended (1×10^6^ cells/mL) in binding buffer (10 mmol/L HEPES, pH 7.4, 140 mmol/L NaCl, 2.5 mmol/L CaCl_2_). MM cells (1×10^5^) were incubated with annexin V-FITC (5 µL; Pharmingen, San Diego, CA) and PI (5 mg/mL) for 15 minutes at room temperature. AnnexinV+PI- apoptotic cells were enumerated by using the flow cytometer (FACS Navios, Beckman Coulter).

### HDAC activity assay

HDAC Activity was performed using the colorimetric HDAC activity assay from BioVision (BioVision Research Products, Mountain View, CA, USA) according to manufacturer instructions. Briefly, 100 µg of nuclear extracts from tumors were diluted in 85 µL of ddH_2_O; then, 10 µL of 10×HDAC assay buffer were added followed by addition of 5 µL of the colorimetric substrate exposed to different concentration of As_2_O_3_; samples were incubated at 37° for 30 min. Subsequently, the reaction was stopped by adding 10 µL of lysine developer and left for additional 30 min at 37°C. Samples were read in a fluorescence plate reader with Ex. = 360 nm and Em. = 460 nm. Inhibition of HDAC activity was expressed as inhibition of Relative Fluorescence Units.

### Western blot analysis

Cell lysates and total protein concentration was measured with the BCA Protein Assay Kit (Pierce Biotechnology, Rockford IL, USA).Equal amounts of protein were subjected to SDS-PAGE and proteins were transferred to nitrocellulose membranes (GE Healthcare, USA). The membrane was blocked in PBS containing 5% non-fat milk and 0.1% Tween-20, washed twice in PBS, and incubated with primary antibody at room temperature for 2 hours, followed by incubation with secondary antibody at room temperature for 45 minutes. Afterward, the proteins of interest were visualized using ECL chemiluminescence system (Santa Cruz Biotechnology, USA).

### Immunoprecipitation

To assess the effects of As_2_O_3_ on Hsp90 acetylation and the Hsp90-IKK interaction, cells (5×10^6^) were treated with As_2_O_3_ at 0 µM, 2.5 µM or 5 µM for 48 hours. Cells were lysed with cell lysis buffer [50 mmol/L Tris-HCl (pH 8.0), 150 mmol/L NaCl, 5 mmol/L EDTA, and 1% Triton X-100] or radioimmunoprecipitation assay buffer [50 mmol/L Tris (pH 8.0), 150 mmol/L NaCl, 0.5% deoxycholate, 0.1% SDS, and 1.0% NP-40] containing 1× protease inhibitor cocktail (Roche, Switzerland) at 4°C with gentle rocking.

The supernatant was precleared to block nonspecific binding with 20 µL protein A-Agarose beads and 20 µL protein G-Agarose beads (Sigma, USA) that had been prewashed three times with RIPA buffer before use. Precleared cellular extract was evenly transferred to 2 new 1.5 mL microcentrifuge tubes. Anti-Hsp90 antibody (2 µL) was added to one microcentrifuge tube and the same amount of normal preimmune IgG from the same origin was added to another tube. After 2 hours incubation at 4°C, 20 µL prewashed protein A-agarose beads and 20 µL prewashed protein G-agarose beads were added to each tube and immunoprecipitation was performed by rocking overnight at 4°C. The immunoprecipitates were eluted by the 2×SDS sample buffer and 1% of input and 20% of bound fractions were resolved by SDS PAGE for western blot analysis with IKKα antibody (Imgenex) and an acetylated lysine Hsp90 antibody (Upstate Biotechnology, Lake Placid, NY) as described above.

### Statistical analysis

Experiments were repeated minimum 3 times with consistent results. Data are expressed as the mean plus or minus SD. Analysis of statistical significance between groups was made using a 2-tailed unpaired Student's t test. A value of *p*<0.05 was considered statistically significant.

## References

[1] Wang ZY, Chen Z (2008). Acute promyelocytic leukemia: from highly fatal to highly curable.. Blood.

[2] Sanz MA, Lo-Coco F (2011). Modern approaches to treating acute promyelocytic leukemia.. J Clin Oncol.

[3] Emadi A, Gore SD (2010). Arsenic trioxide - An old drug rediscovered.. Blood Rev.

[4] Qazilbash MH, Saliba RM, Nieto Y, Parikh G, Pelosini M (2008). Arsenic trioxide with ascorbic acid and high-dose melphalan: results of a phase II randomized trial.. Biol Blood Marrow Transplant.

[5] Vey N, Bosly A, Guerci A, Feremans W, Dombret H (2006). Arsenic trioxide in patients with myelodysplastic syndromes: a phase II multicenter study.. J Clin Oncol.

[6] Sharma M, Khan H, Thall PF, Orlowski RZ, Bassett RL (2011). A randomized phase 2 trial of a preparative regimen of bortezomib, high-dose melphalan, arsenic trioxide, and ascorbic acid..

[7] Miller WH, Schipper HM, Lee JS, Singer J, Waxman S (2002). Mechanisms of action of arsenic trioxide.. Cancer Res.

[8] Hayashi T, Hideshima T, Akiyama M, Richardson P, Schlossman RL (2002). Arsenic trioxide inhibits growth of human multiple myeloma cells in the bone marrow microenvironment.. Mol Cancer Ther.

[9] Beauchamp EM, Ringer L, Bulut G, Sajwan KP, Hall MD (2011). Arsenic trioxide inhibits human cancer cell growth and tumor development in mice by blocking Hedgehog/GLI pathway.. J Clin Invest.

[10] Dos SGA, Abreu ELRS, Pestana CR, Lima AS, Scheucher PS (2011). (+)alpha-Tocopheryl succinate inhibits the mitochondrial respiratory chain complex I and is as effective as arsenic trioxide or ATRA against acute promyelocytic leukemia in vivo..

[11] Lemoine M, Younes A (2010). Histone deacetylase inhibitors in the treatment of lymphoma.. Discov Med.

[12] Aldana-Masangkay GI, Sakamoto KM (2011). The role of HDAC6 in cancer.. J Biomed Biotechnol.

[13] Gregoretti IV, Lee YM, Goodson HV (2004). Molecular evolution of the histone deacetylase family: functional implications of phylogenetic analysis.. J Mol Biol.

[14] Verdin E, Dequiedt F, Kasler HG (2003). Class II histone deacetylases: versatile regulators.. Trends Genet.

[15] Tokesi N, Lehotzky A, Horvath I, Szabo B, Olah J (2010). TPPP/p25 promotes tubulin acetylation by inhibiting histone deacetylase 6.. J Biol Chem.

[16] Valenzuela-Fernandez A, Cabrero JR, Serrador JM, Sanchez-Madrid F (2008). HDAC6: a key regulator of cytoskeleton, cell migration and cell-cell interactions.. Trends Cell Biol.

[17] Yao YL, Yang WM (2011). Beyond histone and deacetylase: an overview of cytoplasmic histone deacetylases and their nonhistone substrates.. J Biomed Biotechnol.

[18] Hinz M, Broemer M, Arslan SC, Otto A, Mueller EC (2007). Signal responsiveness of IkappaB kinases is determined by Cdc37-assisted transient interaction with Hsp90.. J Biol Chem.

[19] Qing G, Yan P, Xiao G (2006). Hsp90 inhibition results in autophagy-mediated proteasome-independent degradation of IkappaB kinase (IKK).. Cell Res.

[20] Gupta SC, Sundaram C, Reuter S, Aggarwal BB (2010). Inhibiting NF-kappaB activation by small molecules as a therapeutic strategy.. Biochim Biophys Acta.

[21] Hayden MS, Ghosh S (2008). Shared principles in NF-kappaB signaling.. Cell.

[22] Bohuslav J, Chen LF, Kwon H, Mu Y, Greene WC (2004). p53 induces NF-kappaB activation by an IkappaB kinase-independent mechanism involving phosphorylation of p65 by ribosomal S6 kinase 1.. J Biol Chem.

[23] Waxman S, Anderson KC (2001). History of the development of arsenic derivatives in cancer therapy.. Oncologist.

[24] Kajiguchi T, Yamamoto K, Iida S, Ueda R, Emi N (2006). Sustained activation of c-jun-N-terminal kinase plays a critical role in arsenic trioxide-induced cell apoptosis in multiple myeloma cell lines.. Cancer Sci.

[25] Wen J, Cheng HY, Feng Y, Rice L, Liu S (2008). P38 MAPK inhibition enhancing ATO-induced cytotoxicity against multiple myeloma cells.. Br J Haematol.

[26] Roussel RR, Barchowsky A (2000). Arsenic inhibits NF-kappaB-mediated gene transcription by blocking IkappaB kinase activity and IkappaBalpha phosphorylation and degradation.. Arch Biochem Biophys.

[27] Kapahi P, Takahashi T, Natoli G, Adams SR, Chen Y (2000). Inhibition of NF-kappa B activation by arsenite through reaction with a critical cysteine in the activation loop of Ikappa B kinase.. J Biol Chem.

[28] Nasr R, Rosenwald A, El-Sabban ME, Arnulf B, Zalloua P (2003). Arsenic/interferon specifically reverses 2 distinct gene networks critical for the survival of HTLV-1-infected leukemic cells.. Blood.

[29] Drobna Z, Jaspers I, Thomas DJ, Styblo M (2003). Differential activation of AP-1 in human bladder epithelial cells by inorganic and methylated arsenicals.. FASEB J.

[30] Davison K, Mann KK, Waxman S, Miller WH (2004). JNK activation is a mediator of arsenic trioxide-induced apoptosis in acute promyelocytic leukemia cells.. Blood.

[31] Sharma V, Koul N, Joseph C, Dixit D, Ghosh S (2010). HDAC inhibitor, scriptaid, induces glioma cell apoptosis through JNK activation and inhibits telomerase activity.. J Cell Mol Med.

[32] Baysan A, Yel L, Gollapudi S, Su H, Gupta S (2007). Arsenic trioxide induces apoptosis via the mitochondrial pathway by upregulating the expression of Bax and Bim in human B cells.. Int J Oncol.

[33] Zhou L, Jing Y, Styblo M, Chen Z, Waxman S (2005). Glutathione-S-transferase pi inhibits As2O3-induced apoptosis in lymphoma cells: involvement of hydrogen peroxide catabolism.. Blood.

[34] Kawaguchi Y, Kovacs JJ, McLaurin A, Vance JM, Ito A (2003). The deacetylase HDAC6 regulates aggresome formation and cell viability in response to misfolded protein stress.. Cell.

[35] Hubbert C, Guardiola A, Shao R, Kawaguchi Y, Ito A (2002). HDAC6 is a microtubule-associated deacetylase.. Nature.

[36] Wu KL, Beksac M, van DJ, Amadori S, Zweegman S (2006). Phase II multicenter study of arsenic trioxide, ascorbic acid and dexamethasone in patients with relapsed or refractory multiple myeloma.. Haematologica.

[37] Boyault C, Sadoul K, Pabion M, Khochbin S (2007). HDAC6, at the crossroads between cytoskeleton and cell signaling by acetylation and ubiquitination.. Oncogene.

[38] Criollo A, Senovilla L, Authier H, Maiuri MC, Morselli E (2010). The IKK complex contributes to the induction of autophagy.. EMBO J.

[39] Gao YS, Hubbert CC, Lu J, Lee YS, Lee JY (2007). Histone deacetylase 6 regulates growth factor-induced actin remodeling and endocytosis.. Mol Cell Biol.

[40] Karin M (2006). Nuclear factor-kappaB in cancer development and progression.. Nature.

[41] Thu YM, Richmond A (2010). NF-kappaB inducing kinase: a key regulator in the immune system and in cancer.. Cytokine Growth Factor Rev.

[42] Sakurai H, Suzuki S, Kawasaki N, Nakano H, Okazaki T (2003). Tumor necrosis factor-alpha-induced IKK phosphorylation of NF-kappaB p65 on serine 536 is mediated through the TRAF2, TRAF5, and TAK1 signaling pathway.. J Biol Chem.

